# I BRAZILIAN CONSENSUS FOR MULTIMODAL TREATMENT OF COLORECTAL LIVER METASTASES

**DOI:** 10.1590/S0102-6720201500040001

**Published:** 2015

**Authors:** Felipe José Fernandez COIMBRA, Heber Salvador de Castro RIBEIRO, Orlando Jorge Martins TORRES

**Affiliations:** 1 A.C. Camargo Cancer Center, São Paulo, Brazil; 2 Federal University of Maranhão, São Luís, Brazil

Liver metastases are a frequent event in the course of colorectal cancer and some studies indicate them as the cause of two thirds of deaths from this disease. Treatment is complex and involves a range of therapeutic options that vary through behavior and timing of metastases diagnosis, as well as the background of the patient. The volume of medical knowledge published every year on this topic is on the rise, with information with different levels of evidence, coming as a torrent of data that daily challenges those involved in the care of these patients. These professionals, in turn, should belong to multidisciplinary teams able to evaluate together the best treatment options as well as their sequence, according to the specifics of each case. 

These are the premises that supported the initiative to hold the **First Brazilian Consensus of Multimodal Treatment of Liver Metastases from Colorectal Cancer**, including, unprecedentedly in Brazil, the specialty societies involved in this care, namely the Brazilian Chapter of the International Hepato-Pancreato Biliary Association (BC-IHPBA), the Brazilian Society of Surgical Oncology (BSSO), the Brazilian Society of Clinical Oncology (BSCO), the Brazilian College of Digestive Surgery (BCDS) and the Brazilian College of Surgeons (BCS), also relying on the support of the Americas Hepato-Pancreato-Biliary Association (AHPBA). Experts from other areas were also involved in specific points of the discussion, such as radiologists, interventional radiologists and pathologists. The Consensus meeting was held on August 23, 2014 during the **X International Symposium on Cancer of the Digestive Apparatus of CEPGIO / International Symposium BC-IHPBA / Postgraduate Course of AHPBA** at A.C. Camargo Cancer Center in São Paulo. 

To organize the flow of ideas and make the practical application of the Consensus a reality, the theme was divided into modules on specific topics:

Module 1: Pretreatment Assessment

Topic 1. Epidemiology and results of treatment in LMCRC

Topic 2. Radiologic and staging diagnosis

Topic 3. Definition of resectability

Topic 4., Clinicopathological and molecular prognostic factors relevant in the definition of treatment

Module 2: Management of resectable metastases 

Topic 5. Management of synchronous resectable disease

Topic 6. Management of metachronous resectable disease

Topic 7. Missing metastases - what to do? 

Module 3: Controversies and unresectable metastases

Topic 8. Treatment in post-chemotherapy progression in resectable disease 

Topic 9. Treatment when extrahepatic disease is found

Topic 10. Conversion treatment in unresectable disease

Topic 11. Palliative treatment

The debates were conducted in two phases. In the Pre-consensus Phase, Specialist**Committees** were established with 4 components each, all members of the supporting societies with recognized competence and practice on the subject, one of which being responsible for submitting data on the day of the Consensus. 

Each committee was responsible for one of the topics of the Consensus and expected to answer the questions elaborated by the organizing committee, systematically reviewing the literature and producing a presentation that synthesized the most relevant data, including up to five recommendations at the end of each topic.

This pre-Consensus phase preceded the proper presentation by two months, with an average of four rounds of discussions between the Specialist Committees and the Organizing Committee, in order to meet all the data raised in the planning of the Consensus. 

For the Attendance Phase, which took place during the CEPGIO symposium, Expert Committees for each Consensus Module were made, again relying on expert professionals who were indicated by the supporting societies, and in collaboration with guests from some of the most renowned cancer treatment institutions in the world. Each Expert Committee discussed and buoyed the presentations and recommendations of the Specialist Committees that had previously been voted on by the present audience. This vote was held after each presentation, and the computed results based on the percentage of agreement submitted for each recommendation sentence: 

Agreement: > 75% of agreement

Partial agreement: 50 - 74% agreement

Disagreement: 0 - 49% agreement

After the Attendance Phase, each Specialist Committee produced a synthesis text of their presentation, including recommendations of their topics with the suggested changes in the debate with the Experts and with the audience. The three following articles presented (the first in this issue and the others in subsequent issues) are intended to make the data available for viewing. We sincerely hope that all this effort will facilitate the arduous task of caring for and each time producing better results in the management of patients with liver metastases from colorectal cancer. 

